# Evaluation of contrast-enhanced ultrasound for activity of rheumatoid arthritis: A protocol for systematic review and meta-analysis

**DOI:** 10.1097/MD.0000000000024417

**Published:** 2021-02-05

**Authors:** Yunpei Zhu, Ping Sui, Cong Wang, Hui Wang, Zhihong Wen

**Affiliations:** a Ultrasound Department of The First Affiliated Hospital of Dalian Medical University.; b Radiology Department of Dalian Fifth People's Hospital.

**Keywords:** contrast-enhanced ultrasound, meta-analysis, rheumatoid arthritis

## Abstract

**Background:**

Contrast-enhanced ultrasound (CEUS) refers to a technique that uses contrast medium to strengthen the echo of backscatter, which can significantly improve the resolution, sensitivity and specificity of ultrasound diagnosis. As a quantitative imaging examination of blood flow signals, CEUS has allowed detection of synovial microvascularization in the joints of patients with rheumatoid arthritis (RA). However, the results of these studies have been contradictory. Therefore, the purpose of this study is to evaluate the value of CEUS in the activity of RA disease.

**Methods:**

We will search PubMed, Embase, Cochrane Library, and CNKI from their inception to the December 20, 2020, without restrictions of language and publication status. Two investigators will independently carry out searching literature records, scanning titles and abstracts, full texts, collecting data, and assessing risk of bias. This study will only include high quality clinical cohort or case control studies. Statistical analysis was performed by using the Review Manager version 5.3 and the STATA version 14.0 (Stata Corp, College Station, TX, USA) softwares.

**Results:**

This systematic review will determine the value of CEUS in RA activity scores.

**Conclusion:**

The results of this study will provide a useful basis for high-quality CEUS to evaluate RA activity score.

**Systematic review registration:**

INPLASY2020120125.

## 1 Introduction

RA is a chronic systemic autoimmune disease, which mainly invades multiple peripheral synovial joints all over the body.^[1]^ The primary pathological features of RA are erosive synovitis, synovial hyperplasia and pannus formation.^[2]^ Synovial lesions occur in the early stage, and hyperemia and edema can be seen in the synovium. Accurate evaluation of synovitis is considerably vital for the therapy of RA, especially for early detection and evaluation of RA during follow-up.^[3]^ The richness of blood flow signals can reflect the severity of RA disease, so as to evaluate the development of RA disease.^[4]^ Therefore, we performed a meta-analysis of high-quality clinical cohort or case-control studies to determine the value of contrast-enhanced ultrasound in evaluating joint activity in RA.

## 2 Materials and methods

The protocol has been performed according to the PRISMA (preferred reporting project for systematic review and meta-analysis) guidelines and it has been registered in the INPLASY (INPLASY2020120125).

### 2.1 Eligibility criteria

#### 2.1.1 Participants

All patients were in accordance with diagnostic criteria of RA established by the American College of Rheumatology (ACR) in 1987 or the European alliance against Rheumatism (EULAR)/ACR in 2009 or the Chinese institute of Rheumatology in 2010 or the World Health Organization (WHO) in 2008 in spite of race, nationality, and sex.^[5]^

#### 2.1.2 Intervention and comparison

All patients were assessed with CEUS and Laboratory examination.

#### 2.1.3 Outcomes

The primary outcomes include a quantitative scoring system, through which synovial vascularity intensity was evaluated by means of CEUS.

#### 2.1.4 Type of study

This study will only include high quality clinical cohort or case control studies.

### 2.2 Search methods

PubMed, Embase, Cochrane Library, and CNKI will be searched to identify relevant studies from their inception to the December 20, 2020, without restrictions of language and publication status. The search strategy for PubMed is shown in Table 1. The similar search strategy will also used to other databases.

**Table 1:** Search strategy sample of pubmed.

Number	Search terms
1	contrast enhanced ultrasound OR contrast enhanced ultrasonographyOR contrast enhanced sonography OR contrast enhanced echographyOR contrast enhanced echotomography ORcontrast enhanced ultrasonic diagnosis OR CEUS
2	rheumatoid arthritis OR RA
3	#1 AND #2

### 2.3 Data extraction and quality assessment

Two investigators will independently extract the essential data from all included studies. The following data were extracted from the eligible studies: year of article, the first authors surname, sample size, examination position, mean of disease activity score of 28 joints, Instrument. The methodological quality was independently assessed by 2 investigators according to the methodological index for nonrandomized studies (MINORS). The MINORS criteria included 12 assessment items and each of these items was scored as “yes” (2), “no” (0), or “unclear”. MINORS score ranged from 0 to 24; and score ≥ 13 indicated a good quality. Any disagreements between 2 investigators will be solved through discussion or consultation by a third investigator.

### 2.4 Statistical analysis

The Review Manager version 5.3 and the STATA version 14.0 (Stata Corp, College Station, TX, USA) softwares were used to perform meta-analysis of the pooled data. We calculated the pooled summary odds ratio (OR) and its 95% confidence interval (CI). The Cochrane Q-statistic and *I*^2^ test were used to evaluate potential heterogeneity between studies. If Q test shows a *P* < .05 or *I*^2^ test exhibits >50% which indicates heterogeneity, the random-effect model was conducted, or else the fixed-effects model was used. In order to evaluate the influence of single study on the overall estimate, sensitivity analysis was performed. In addition, Subgroup and meta-regression analyses were performed to distinguish the potential sources of heterogeneity between studies. Moreover, both the Begg funnel plot and Egger test were applied to evaluate potential publication bias.^[6]^

### 2.5 Ethics and dissemination

The data of our study will be obtained from published literature, so ethical approval will be not required. We expect to publish this study on a peer-reviewed journal.

## 3 Discussion

The degree of synovial congestion in patients with RA is closely related to the activity of the disease, which can be used as an important auxiliary index to judge the activity of synovium.^[7–10]^ Studies have shown that synovial thickness after radiography can reflect the inflammatory activity of RA, and can compare its changes in the process of disease follow-up and dynamic observation to reflect the disease activity, and provide a vital basis for guiding clinical therapy.^[11]^ However, the results of these studies have been contradictory. To gain clarity, in this study, we will conduct a systematic review to summarize high-quality studies and to provide evidence on the evidence-based medical support for clinical practice.

## Author contributions

**Conceptualization:** Cong Wang, Hui Wang.

**Data curation:** Yunpei Zhu, Ping Sui.

**Methodology:** Yunpei Zhu, Ping Sui.

**Writing – original draft:** Yunpei Zhu, Ping Sui.

**Writing – review & editing:** Yunpei Zhu, Ping Sui, Hui Wang, Zhihong Wen.
